# Comparison of LED and Conventional Fluorescence Microscopy for Detection of Acid Fast Bacilli in a Low-Incidence Setting

**DOI:** 10.1371/journal.pone.0022495

**Published:** 2011-07-21

**Authors:** Jessica Minion, Madhukar Pai, Andrew Ramsay, Dick Menzies, Christina Greenaway

**Affiliations:** 1 Department of Epidemiology, Biostatistics and Occupational Health, McGill University, Montreal, Canada; 2 Respiratory Epidemiology and Clinical Research Unit, Montreal, Canada; 3 Department of Medical Microbiology and Immunology, University of Alberta, Edmonton, Canada; 4 UNICEF/UNDP/World Bank/WHO Special Programme for Research and Training in Tropical Diseases, World Health Organization, Geneva, Switzerland; 5 Department of Diagnostic Medicine, Division of Infectious Diseases, SMBD-Jewish General Hospital, McGill University, Montreal, Canada; National Institute for Infectious Diseases (L. Spallanzani), Italy

## Abstract

**Introduction:**

Light emitting diode fluorescence microscopes have many practical advantages over conventional mercury vapour fluorescence microscopes, which would make them the preferred choice for laboratories in both low- and high-resource settings, provided performance is equivalent.

**Methods:**

In a nested case-control study, we compared diagnostic accuracy and time required to read slides with the Zeiss PrimoStar iLED, LW Scientific Lumin, and a conventional fluorescence microscope (Leica DMLS). Mycobacterial culture was used as the reference standard, and subgroup analysis by specimen source and organism isolated were performed.

**Results:**

There was no difference in sensitivity or specificity between the three microscopes, and agreement was high for all comparisons and subgroups. The Lumin and the conventional fluorescence microscope were equivalent with respect to time required to read smears, but the Zeiss iLED was significantly time saving compared to both.

**Conclusions:**

Light emitting diode microscopy should be considered by all tuberculosis diagnostic laboratories, including those in high income countries, as a replacement for conventional fluorescence microscopes. Our findings provide support to the recent World Health Organization policy recommending that conventional fluorescence microscopy be replaced by light emitting diode microscopy using auramine staining in all settings where fluorescence microscopy is currently used.

## Introduction

Tuberculosis (TB) continues to be one of the world's most important infectious causes of morbidity and mortality. An estimated 9.4 million people develop TB disease each year and approximately 1.7 million die from the disease [1]. While the preponderance of TB burden is borne by nations in Asia and Africa, TB remains an important public health concern in high-income as well as low- and middle-income countries globally [2].

One of the key steps in TB control is case detection. Although advances in diagnostics are leading to the introduction of new tests [3], [4], the backbone of TB diagnosis worldwide continues to be smear microscopy. Thus, increasing the sensitivity of smear microscopy could have a large impact on global TB case detection rates. As a result there have been several initiatives to optimise smear microscopy including changes in specimen collection procedures, specimen processing, and microscopy techniques [5], [6], [7].

For microscopic detection of acid fast bacilli (AFB), fluorescence microscopy (FM) using auramine staining has been shown to have 10% higher sensitivity compared to routine light microscopy used with Ziehl-Neelsen (ZN) staining, without compromising specificity [8]. FM is also more time efficient, with one large study reporting FM to take only 25% of the time required for ZN examinations [9]. In most high-income countries, FM has now been widely adopted and is used routinely.

Light emitting diode (LED) microscopy is a novel diagnostic tool developed primarily to allow resource-poor parts of the world access to the benefits of FM [10], [11], [12]. Compared to conventional mercury vapour fluorescence microscopes, LED microscopes are less expensive and have lower maintenance requirements. The diodes are very durable, do not require warm-up time, and do not contain toxic products. Importantly, they are reported to perform equally well without a darkroom. These qualities make them attractive for use in low- and middle-income countries, and they have performed well in evaluations in these settings [13], [14], [15], [16], [17], [18], [19], [20]. Many of the benefits of LED technology would also be appealing to laboratories in high-income countries if LED microscopy is equivalent in performance to conventional FM. Indeed, the World Health Organization (WHO) recently recommended that conventional FM be replaced by LED-FM in all settings where fluorescence microscopy is currently used, and that LED FM be phased in as an alternative to conventional ZN microscopy in all settings [21]. Despite this recommendation, this is the first evaluation of LED-FM for TB diagnosis based in a low-burden, high-resource setting.

The objectives of this study were to compare the sensitivity and specificity of fluorescence smear microscopy in the detection of AFB using two different LED microscopes: the Lumin Portable Fluroescence Kit (LW Scientific , Atlanta, Georgia, USA) and the PrimoStar iLED (Carl Zeiss MicroImaging, Jena, Germany), with a conventional mercury vapour fluorescence microscope, using mycobacterial culture as a reference standard, in a low-incidence setting. Additionally, we compared the time required to read auramine-stained smears with the three FM devices and collected feedback from microscopists regarding user-important characteristics.

## Methods

### Study Setting

This study was conducted in Montreal, Quebec, Canada using specimens submitted routinely for mycobacterial culture from university and tertiary care centres, from April through September 2009. These included both diagnostic and follow-up specimens. In 2009 the province of Quebec reported 195 new and retreatment cases, with an incidence rate of 2.5/100,000 [2]. The prevalence of HIV infection in Canada is approximately 0.2% [22]. Overall culture positivity in specimens submitted from patients with suspected mycobacterial disease was approximately 2% with about 40% of these consisting of non-tuberculous mycobacteria (NTM).

### Specimen Selection

Given the low culture positivity rate in our setting, we elected to use a nested case control design so as to include all culture positive specimens and an equal number of culture negative specimens. All consecutive specimens submitted for mycobacterial culture had an additional smear prepared and heat fixed. These unstained smears were stored in dry, dark smear boxes. When culture results became available, all smears originating from culture positive specimens were selected for study inclusion. An equal number of smears originating from culture negative specimens were randomly selected (using random number generators).

### Fluorescence Microscopy Comparison

The Zeiss PrimoStar iLED microscope (Carl Zeiss MicroImaging GmbH, Jena, Germany) was developed in collaboration with FIND (Foundation for Innovative New Diagnostics) and is a stand-alone microscope that can be used in bright-field or fluorescence LED modes [23]. The Lumin Portable Fluorescent Kit (LW Scientific, Atlanta, Georgia, USA) is a portable objective lens attachment that is used with an existing light microscope (in this study it was used with the Zeiss iLED) [24]. We used both the 20x and 40x lenses, the latter for screening and the former for AFB confirmation. We chose to evaluate these two devices upon consideration of their suitability to high-resource settings (Zeiss), their unique benefits compared to conventional fluorescent microscopes (Lumin), and their current popularity in global evaluations (Zeiss and Lumin).

All smear examinations were done by one of two technologists with expertise in mycobacteriology and fluorescence microscopy, who were blinded to the culture results and any patient details. Smears were examined three times by the same microscopist using the LW Scientific Lumin LED attachment, the Zeiss Primo Star iLED, and a conventional mercury vapour microscope (Leica DMLS). Between readings slides were randomized (with the aid of random number lists) to maintain technologist blinding. Staining of slides and readings with all three microscopes were done on the same day to avoid possible fading of the fluorescent stain [25].

Slides were reported as doubtful, 1+, 2+, 3+, 4+ or negative at 400x magnification [26]. Negative smears had 300 fields examined before being declared negative. The time required to read slides was estimated by logging the time at the start and at the end of reading a group of slides. This time was then averaged for the number of slides read to calculate a time per slide estimate, which included mounting slides and recording results.

### Specimen Processing

Respiratory specimens (including sputum, bronchioalveolar lavage (BAL), bronchial wash (BW), lung aspirates) underwent digestion/decontamination with NALC-NaOH and concentration using centrifugation before slide preparation and culture inoculation. Extrapulmonary specimens were processed using standard procedures [26].

Each specimen was inoculated onto Lowenstein-Jensen (LJ) media and into a MGIT tube (Becton Dickenson, Sparks, MD, USA) for up to 8 weeks. Positive growth was confirmed by Kinyoun staining, and mycobacterial isolates were sent to the Quebec Provincial Laboratory for speciation using 16S ribosomal sequencing.

Smears were heat-fixed before storage. Staining was performed immediately before the first smear examination. Smears were flooded with auramine O for 15 minutes, then rinsed with sterile water; decolourized with acid-alcohol for 2 minutes, then rinsed with sterile water; counterstained with potassium permanganate for 2-4 minutes, then rinsed with sterile water and allowed to air dry.

### Analysis

Sensitivity, specificity, positive and negative predictive values, and likelihood ratios were calculated for the LW Scientific Lumin LED attachment, the Zeiss Primo Star iLED and conventional mercury vapour (Leica DMLS) microscope using mycobacterial culture as the reference standard. Confidence intervals were constructed using exact methods for proportions [27]. Yield was calculated using a second reference standard where any slide read as positive using any microscope was considered positive. Agreement between the three microscopy readings was estimated using un-weighted kappa statistics (with results dichotomized as positive or negative), as well as weighted kappa statistics with linear weighting of 5 categories: negative, 1+, 2+, 3+, and 4+. Kappa statistics were used to measure the agreement between readings made using two microscopes, with the same reader evaluating the same mycobacterial smears, while taking into account the agreement occurring by chance [28].

Subgroup analysis was done by specimen type (sputum, non-sputum respiratory, extrapulmonary) and mycobacterial species isolated (*M. tuberculosis* complex, non-tuberculous mycobacteria and acid fast non-mycobacteria).

## Results

A total of 200 culture positive specimens were included in the study, with 200 randomly selected culture-negative controls. 296 specimens were submitted as sputum (74.0%), 64 originated from the respiratory system but not classified as sputum (16.0%; includes specimens such as BAL fluid, BW and lung biopsies), and 40 specimens were categorized as extrapulmonary (10.0%).

Using mycobacterial culture as a reference standard, the accuracy of the 3 microscopes is shown in Table 1. Zeiss achieved the highest sensitivity with 40.5% (95% CI: 33.6, 47.7), followed by Lumin with 37.5% (95% CI: 30.8, 44.6) and conventional fluorescence microscope with 36.5% (95% CI: 29.8, 43.6). None of the differences in sensitivity were significantly different based on overlapping confidence intervals. Specificity was very similar between all 3 microscopes (conventional fluorescence microscope and Zeiss were equal: 99.0% [95% CI: 96.4, 99.9]; and Lumin: 99.5% [95% CI: 97.2, 100]).

**Table 1:** Accuracy Using a Culture Reference Standard.

	TP/Cx+	Sensitivity (95% CI)	TN/Cx-	Specificity (95% CI)
**conventional FM**	73/200	36.5% (29.8, 43.6)	198/200	99.0% (96.4, 99.9)
**Zeiss**	81/200	40.5% (33.6, 47.7)	198/200	99.0% (96.4, 99.9)
**Lumin**	75/200	37.5% (30.8, 44.6)	199/200	99.5% (97.2, 100)

	PPV*	NPV*	LR+	LR-
**conventional FM**	0.97 (0.91, 1.00)	0.61 (0.55, 0.66)	36.5 (9.1, 146.7)	0.6 (0.6, 0.7)
**Zeiss**	0.98 (0.92, 1.00)	0.62 (0.57, 0.68)	40.5 (10.1, 162.5)	0.6 (0.5, 0.7)
**Lumin**	0.99 (0.93, 1.00)	0.61 (0.56, 0.67)	75.0 (10.5, 534.2)	0.6 (0.6, 0.7)

TP  =  true positive, TN  =  true negative, Cx +  =  culture positive, Cx −  =  culture negative, PPV  =  positive predictive value, NPV  =  negative predictive value, LR+  =  positive likelihood ratio, LR-  =  negative likelihood ratio.

*PPV and NPV calculated for fixed prevalence of 50% due to case-control study design.

There were 87 specimens which were read as smear positive by at least 1 of the 3 microscopes. The conventional fluorescence microscope identified 75 specimens as smear positive, the Zeiss identified 83 and the Lumin identified 76. Using a reference standard where any positive microscopic reading was considered a true positive, this resulted in sensitivities of 86.2% (95% CI: 77.1, 92.7), 95.4% (95% CI: 88.6, 98.7), and 87.4% (95% CI: 78.5, 93.5) for conventional fluorescence microscope, Zeiss and Lumin respectively (Table 2).

**Table 2:** Sensitivity Using a Microscopic Reference Standard.

	Smear + / “Any Smear Positive”	Sensitivity (95% CI)
**conventional FM**	75/87	86.2% (77.1, 92.7)
**Zeiss**	83/87	95.4% (88.6, 98.7)
**Lumin**	76/87	87.4% (78.5, 93.5)

**Figure 1:**
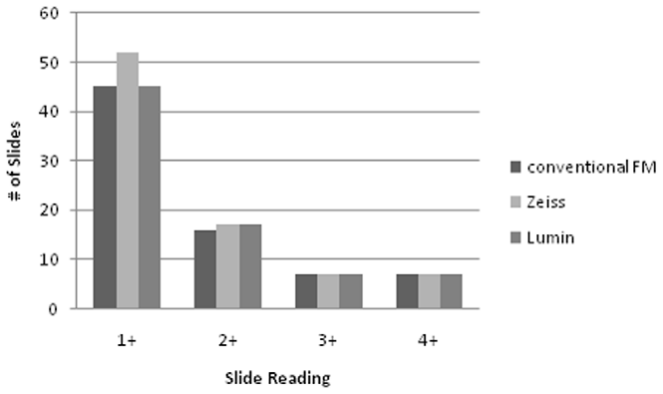
Distribution of positive smear readings. *No doubtful/scanty results were reported.

Agreement was measured with the kappa statistic using dichotomized results (where 1+, 2+, 3+ and 4+ were pooled as positive). Agreement was high between all three microscopes: unweighted kappa  = 0.91 (95% CI: 0.85, 0.96) between conventional fluorescence microscope and Zeiss; 0.89 (95% CI: 0.84, 0.95) between conventional fluorescence microscope and Lumin; and 0.91 (95% CI: 0.86, 0.96) between Zeiss and Lumin. Kappa values remained high if linear weights for categories of smear positivity (negative, +1, +2, +3, +4) were used: weighted kappa  = 0.92 (95% CI: 0.89, 0.96) between conventional fluorescence microscope and Zeiss; 0.92 (95% CI: 0.88, 0.96) between conventional fluorescence microscope and Lumin; and 0.93 (95% CI: 0.90, 0.97) between Zeiss and Lumin. The distribution of all positive smear readings is displayed in Figure 1.

Accuracy was also calculated depending on the category of specimens examined and the species isolated. Table 3 shows sensitivity and specificity of all 3 microscopes stratified by sputum specimens, non-sputum respiratory specimens, and extra-pulmonary specimens. There were no significant differences between the microscopes for any of these subgroups, based on non-overlapping confidence intervals.

**Table 3:** Accuracy by Specimen Type.

a) Sputum				
	TP/Cx+	Sensitivity (95% CI)	TN/Cx-	Specificity (95% CI)
**conventional FM**	63/169	37.3% (30.0, 45.0)	125/127	98.4% (94.4, 99.8)
**Zeiss**	68/169	40.2% (32.8, 48.0)	125/127	98.4% (94.4, 99.8)
**Lumin**	64/169	37.9% (30.5, 45.6)	126/127	99.2% (95.7, 100)

b) Other Respiratory*				
	TP/Cx+	Sensitivity (95% CI)	TN/Cx-	Specificity (95% CI)
**conventional FM**	5/17	29.4% (10.3, 56.0)	47/47	100% (92.5, 100)
**Zeiss**	6/17	35.3% (14.2, 61.7)	47/47	100% (92.5, 100)
**Lumin**	6/17	35.3% (14.2, 61.7)	47/47	100% (92.5, 100)

c) Extrapulmonary				
	TP/Cx+	Sensitivity (95% CI)	TN/Cx-	Specificity (95% CI)
**conventional FM**	5/14	35.7% (12.8, 64.9)	26/26	100% (86.8, 100)
**Zeiss**	7/14	50.0% (23.0, 77.0)	26/26	100% (86.8, 100)
**Lumin**	5/14	35.7% (12.8, 64.9)	26/26	100% (86.8, 100)

*includes specimens from respiratory system other than sputum (e.g. BAL, lung biopsy).

TP  =  true positive, TN  =  true negative, Cx +  =  culture positive, Cx -  =  culture negative.

Of the 200 culture positive specimens, MTB Complex organisms were isolated from 115 (106 *M. tuberculosis*, 9 *M. africanum*). The remaining 85 culture positive specimens isolated a wide range of NTM as well as acid-fast organisms capable of surviving mycobacterial decontamination and growing in mycobacterial growth media (2 *Streptomyces* species, 1 *Nocardia puris*, 1 *Tsukamurella tyrosinosolvens*). These were considered true positives since organisms from Streptomyces, Nocardia and Tsukamurella genera are considered acid fast. The sensitivity of all 3 microscopes was higher in detecting MTB Complex organisms compared to NTM or other acid fast organisms; however, there was no difference between the 3 devices (Table 4).

**Table 4:** Sensitivity by Species Isolated.

	MTB Complex^1^		NTM^2^ & others^3^	
	TP/Cx+	Sensitivity (95% CI)	TP/Cx+	Sensitivity (95% CI)
**conventional FM**	57/115	49.6% (40.1, 59.0)	16/85	18.8% (11.2, 28.8)
**Zeiss**	61/115	53.0% (43.5, 62.4)	20/85	23.5% (15.0, 34.0)
**Lumin**	58/115	50.4% (41.0, 59.9)	17/85	20.0% (12.1, 30.1)

1 includes 106 *M. tuberculosis*, 9 *M. africanum.*

2 NTM includes 26 *M. avium*, 16 *M. gordonae*, 7 *M. kansasii*, 7 *M. chimaera*, 6 *M. intracellulaire*, 3 *M. conceptionense*, 2 *M. abscessus*, 2 *M. xenopi*, 2 *M. porcinum*, 2 *M. simiae grp*, 1 *M. fortuitum*, 1 *M. shimoidei*, 1 *M. terrae*, 1 *M. celatum*, 1 *M. lentifalvum*, 3 *Mycobacterium* spp (undetermined).

3 others include 2 *Streptomyces* spp., 1 *Norcardia puris*, 1 *Tsukamurella tyrosinosolvens.*

TP  =  true positive.

Cx+  =  culture positive.

On average, reading slides using the conventional fluorescence microscope took 1.51 mins/slide (95% CI: 1.47, 1.55). This was identical to the time required using the Lumin (1.51 mins/slide; [95% CI: 1.48, 1.54]), but longer than the time required using the Zeiss (1.12 mins/slide; [95% CI: 1.09, 1.15]). The time savings using the Zeiss microscope was statistically significant compared to the other 2 microscopes, based on non-overlapping CIs.

## Discussion

The benefits of improved sensitivity and reading efficiency of FM compared to ZN microscopy have long been realized in high-income countries through the use of conventional mercury vapour fluorescence microscopes. The operational benefits of LED microscopes over conventional fluorescence microscopes would certainly be of interest for laboratories and technologists in high-income as well as low- and middle-income settings. Working without the need for a dark room using LED microscopes could significantly improve workflow and maximize space utilization in the lab, in addition to the benefits seen in tropical climates relating to the absence of climate control in enclosed spaces. Lower purchase price and maintenance costs, longer diode life, absence of toxic components, and the lack of warm up time required between turning on a conventional fluorescence microscope and its use are all factors that would influence laboratories in high-income countries to switch from conventional to LED FM.

However, these benefits would not be sufficient to adopt LED FM in high-income settings unless the sensitivity and reading efficiency associated with conventional FM were maintained with LED FM. Additionally, laboratory technologists in high-income countries are generally familiar with FM already and have ample expertise using conventional fluorescence microscopes. While this obviates the need for extensive training when introducing LED FM, it sets high expectations of device quality and usability.

This is the first study evaluating LED microscopy for AFB detection in a high-income, low-incidence setting, as a practical improvement over existing conventional FM. Just as it is unadvisable to extrapolate results from studies performed in high-income, low-incidence settings to low-income, high-incidence settings, it would be equally inappropriate to make inferences in the opposite direction. Factors such as TB- and HIV-prevalence, disease severity, proportion of non-tuberculous mycobacteria, and type of specimens received will affect the external validity of any TB diagnostic evaluation.

An illustration of this is seen in the diagnostic sensitivity we report for all 3 FM modalities, which is lower than the pooled estimates of sensitivity found in a recent systematic review and meta-analysis (84% sensitivity compared to culture as a reference standard) [29]. This emphasizes a common difference between a high-income, low-incidence setting such as ours and the majority of settings represented in the review. The fact that we have a much higher proportion of smear negative, culture positive specimens will lead to all types of microscopic TB diagnostics appearing to underperform when compared to culture. While the same is often seen with high incidences of TB and HIV co-infection, in this case it is likely a combination of a relatively higher proportion of extra-pulmonary TB and non-tuberculous mycobacterial infections, which are more often smear-negative, as well as health system practices such as the submission of specimens for follow-up of incidental chest x-ray finding, symptom-free immigration screening, active case finding among TB contacts, and generally less advanced disease among those diagnosed.

In this LED evaluation, we found no difference in diagnostic accuracy between the Zeiss Primo Star iLED, the LW Scientific Lumin and the conventional fluorescence microscope (Leica DMSL). The agreement was high for all three microscopes assessed (kappa >0.88 for all comparisons). When smears were stratified by their specimen type or organism isolated, their diagnostic accuracy remained equivalent. However, our evaluation was limited by the small number of culture-positive and smear-positive specimens available for inclusion, resulting in wide confidence intervals around estimates of diagnostic accuracy. The analysis was also performed by specimen and not by patient. While this is consistent with most other studies in this field, we recognize that the lack of independence between specimens arising from the same patient may overestimate the precision of accuracy estimates.

For laboratory managers considering the implementation of LED microscopy in either a low- or high-income setting, the choice of LED device is important. There are several commercial manufacturers now marketing LED microscopes, but few studies comparing their head-to-head performance [15], [30]. Given the wide variety of devices available, each with different benefits claimed and potential roles, it is important to compare them with respect to a specific setting or situation. For instance, in high-income, low-incidence laboratories, portability and the ability to withstand power fluctuations and dusty environments are less important considerations. In comparison, technologist acceptance, speed of reading, and confirmation that readings can be made without a darkroom continue to be important, specifically when compared to currently available high quality mercury vapour fluorescence microscopes.

The time required to examine slides was identical for the conventional fluorescence microscope and the Lumin LED attachment. However, the average time spent examining slides with the Zeiss Primo Star iLED was significantly less than with the other two microscopes. Subjective reports from our technologists confirmed that the Zeiss was the easiest of the 3 to use, provided the most convenient focusing and brightest viewing fields when screening slides. Importantly, the technologists confirmed that the Zeiss microscope was easily used without a darkroom. This was similar to the user -reviews reported by Albert et al. [15].

The spectrum of light produced by LED devices is narrower than that provided by mercury vapour conventional fluorescence microscopes and its wavelength is produced to match specifically the peak absorbance of auramine stains [10]. This likely contributes to the increased brightness produced by LED microscopes and explains why they can be used without a darkroom. However, the Lumin attachment did not demonstrate the same superior reading efficiency as the Zeiss. Our technologists reported that the Lumin was more difficult to focus, and the resulting fluorescence of the auramine-stained bacilli was dim and they would not recommend its use without a darkroom. The manufacturers of the Lumin have since recognized that the objective light source was too dim, and newer models have been improved in this regard. Another practical characteristic of the Lumin (and other similar objective lens attachments) is the fact that the light source needs to be plugged in directly to the objective lens being used. Not only can this create an obstruction while the technologist is working, but it also makes it inconvenient to switch between different objective lenses and thus different viewing magnifications. This assumes you have more than one Lumin objective lens (as we did in this study); otherwise you are strictly limited to one magnification. While the Lumin has received both positive and negative reviews in other studies [13], [15], [18], [30], many of its benefits (including low upfront cost and portability) are less important in most high-income settings.

### Conclusions

The LED fluorescent microscopes (Zeiss Primo Star iLED and LW Scientific Lumin) had nearly identical accuracy compared to a conventional fluorescent microscope (Leica DMLS) for the detection of AFB in patient specimens. The Zeiss required significantly less time for smear examination compared to either the conventional fluorescence microscope or the Lumin. Given the practical benefits of LED microscopes for TB diagnosis, and comparable accuracy to the current standard of a conventional fluorescence microscope, we conclude that LED microscopy should be considered by all TB diagnostic laboratories, including those in high-income settings, as a replacement for conventional FM. Our findings provide support for the recent WHO policy which recommended that conventional FM be replaced by LED FM using auramine staining in all settings where FM is currently used [21].
